# A Low-Cost IEEE 802.15.7 Communication System Based on Organic Photodetection for Device-to-Device Connections

**DOI:** 10.3390/s20030714

**Published:** 2020-01-28

**Authors:** Pablo Corral, Fernando Rodríguez-Mas, José Luis Alonso, Juan Carlos Ferrer, Susana Fernández de Ávila

**Affiliations:** Communications Engineering Department, Universidad Miguel Hernández, Av. Universidad, s/n, Ed. Innova, 03202 Elche, Spain; fernando.rodriguezm@umh.es (F.R.-M.); j.l.alonso@umh.es (J.L.A.); jc.ferrer@umh.es (J.C.F.); s.fdezavila@umh.es (S.F.d.Á.)

**Keywords:** visible light communications, organic photodetector, P3HT:PCBM, IEEE 802.15.7

## Abstract

In this article, we compare two different kinds of commercial light-emitting diodes (LEDs) in transmission and organic photodetectors based on poly(3-hexylthiophene) (P3HT) and a phenyl-C61-butyric acid methyl ester (PCBM) blend used as active layer in reception. Photovoltaic cells based on massive heterojunctions of semiconductor polymers have focused the attention of researchers due to their several potential advantages over their inorganic counterparts, such as their simplicity, low cost, and ability to process large area devices, even on flexible substrates. Furthermore, in logistics, storage management systems require the implementation of technological solutions that allow the control of merchandise in real time by means of light-emitting diode signals that send information about the product. However, the slow response time of these organic photodetectors should not be critical for this application, where the light intensity changes are very slow, which limits the speed of data transmission compared to inorganic based systems that use wireless optical communications. Finally, we show a low-cost visible light communication system based on organic photodetectors with a frame based on on-off keying with Manchester encoding to support device-to-device connections.

## 1. Introduction

In visible light communications (VLC), data are transmitted by intensity modulating optical sources, such as light-emitting diodes (LEDs) and laser diodes (LDs), which occurs faster than the persistence of the human eye [1]. In the IEEE 802.15.7 optical wireless communication (OWC) standard, variable pulse position modulation (VPPM) and on-off keying (OOK) for optical wireless personal area networks (OWPANs) were released for dimming control, and those modulation schemes were defined for a single light source in different physical layers [2]. This standard includes more optical wireless communication technologies and describes the use of OWC for optical wireless personal area networks (OWPANs). Besides, it covers topics such as network topologies, addressing, collision avoidance, acknowledgment, performance quality indication, dimming support, visibility support, coloured status indication, and colour stabilization. In this research, OOK is used with Manchester code for stable dimming support and the avoidance of flickering.

Concerning electronic devices based on organic materials, those related to semiconducting polymers have attracted great interest in the research community over the last two decades, as they appear to be best candidates to replace their inorganic counterparts. This is not only due to fabrication cost reduction, since these polymers can be deposited from solution using easy and low-cost techniques like spin coating, doctor blade, etc., but also for the possibility to easily be set down over large areas, even over flexible substrates.

As a matter of fact, one of the most common kinds of materials used for constructing polymer-based electronic devices are those based on polythiophene derivatives. Among these polythiophenes, poly(3-hexylthiophene)—P3HT stands out due to its excellent electrical properties when embedded in an electronic device. These properties include the high mobility of holes, ranging from 1.33 × 10^−5^ cm^2^V^−1^s^−1^ to 3.30 × 10^−4^ cm^2^V^−1^s^−1^, depending on the molecular weight [3], and the low width of the band gap (1.9 eV) [4]. P3HT has emerged as a good candidate for developing organic photovoltaic cells [5] and organic field effect transistors [6].

Nonetheless, when used in these kinds of devices, P3HT is not used alone, instead, it is usually reported in the specific literature in combination with other compounds, such as phenyl-C_61_-butyric acid methyl ester (PCBM) [7], organic dyes [8], TiO_2_ nanoparticles [9], or carbon nanotubes [10].

So, in this paper, we show a low-cost system based on organic photodetectors using ATmega328 (Elche, Spain), an Atmel 8-bit AVR RISC-based microcontroller, and a frame based on OOK with Manchester coding type 1 to support an OWPAN. Organic photodetectors (OPDs) have been tested for VLC with positive results [11,12,13,14,15]. Our organic photodetector is able to transmit text via VLC.

## 2. Materials and Methods

### 2.1. Description of the System

**Figure 1:**
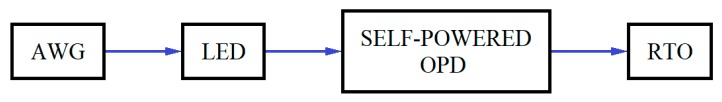
Block diagram of the experimental setup, using an arbitrary waveform generator, a high luminosity light-emitting diode, a self-powered OPD, and a RTO. AWG: Arbitrary waveform generator; RTO: Real-time oscilloscope. LED: Light-emitting diode, OPD: Organic photodetector.

Figure 1 shows the configuration of the experimental setup in order to detect the maximum supported frequency of the VLC system. In this demonstration, the transmitter is a single high luminosity white-light phosphor-based LED chip, with a correlated colour temperature (CCT) of 5500 K. An arbitrary waveform generator (AWG, Tektronix ^®^ AFG320) was used to generate the Manchester coding signals that were applied to the LED. The analogical bandwidth and the sample rate of the AWG were 16 MHz and 16 MSample/s, respectively. The generated VLC signal will travel a fixed transmission distance and then be detected by the self-powered OPD. Then, the self-powered OPD was connected to a real-time oscilloscope (RTO, Tektronix ^®^ TDS3012) for signal detection. The analogical bandwidth and the sampling rate of the RTO were 100 MHz and 1.25 GSample/s respectively.

**Figure 2:**
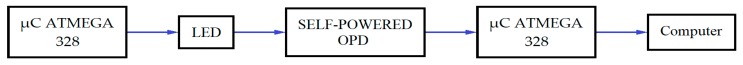
Block diagram of the experimental setup, using a low-cost microcontroller, a high luminosity LED, and the self-powered OPD.

Figure 2 shows the experimental setup of the self-powered OPD-based VLC system used to detect the maximum supported distance of the system. In this experiment, the transmitter was a single high luminosity white-light phosphor-based LED chip, with a correlated colour temperature (CCT) of 5500 K. An ATmega 328 microcontroller was used to generate the Manchester code signals, which were applied to the LED at 1 KHz. The generated VLC signal will travel different transmission distances and then be detected by the self-powered OPD. Then, the self-powered OPD was connected to the ATmega 328 microcontroller for signal detection. All the results were shown on the computer used in the experiment. The clock and sampling rates of the ATmega 328 microcontroller were 16 MHz and 10 k Sample/s, respectively. The difference between the clock frequency and the working frequency is due to the use of certain instructions that have a limitation in their reading cycle, as well as the size of the source code used, and the utilization of some libraries and functions.

ATmega328 is commonly used in systems where a simple, low-power, low-cost micro-controller is needed.

Commercial LEDs were used as light signal emitters. To cover the spectrum, LEDs with different wavelengths have been studied. All commercial LEDs were purchased from Electan and their characterizations were performed with a Triax 190 monochromator and a multichannel thermoelectrically cooled CCD Symphony detector by Horiba Jobin Yvon.

**Figure 3:**
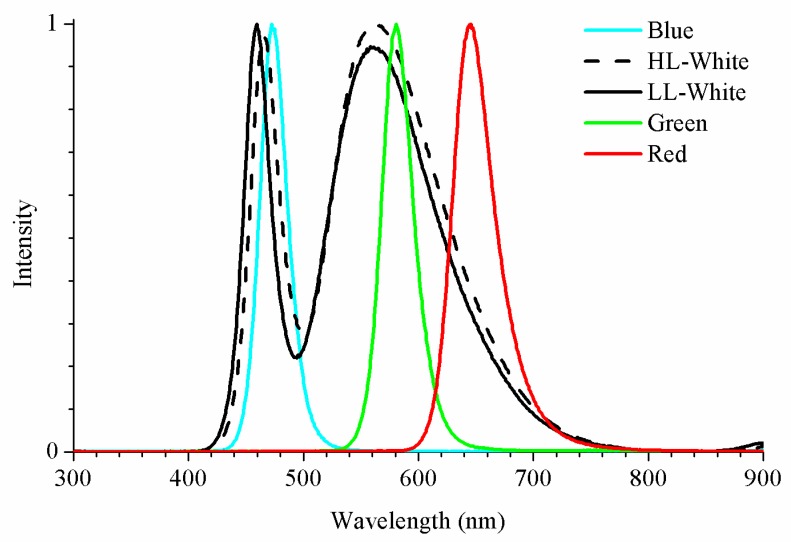
Relative spectral power distribution of the LED used in the experiment.

Figure 3 shows the intensity spectra of the LEDs used in our study. It is noteworthy that two types of white LEDs were employed, high- and low-luminosity white LEDs (HL-white and LL-white). In reviewing the spectra, the wavelengths of the maximum emission peaks of LL-White and HL-White presented very close values, namely, 459 and 465 nm for the narrow peak and 559 and 562 nm for the broad peak (Table 1). Besides, the difference between the full width at half maximum (FWHM) of both spectra was negligible, which was 2 and 4 nm, respectively. Therefore, these may be sufficient reasons to affirm that the white LEDs have the same spectral distribution, although their intensities were different, where the HL-White intensity is double that of LL-White. In order to measure the irradiance, a BPW34 photodetector was used as a reference.

### 2.2. Materials

For the OPD manufacturing process, PEDOT:PSS (poly(2,3-dihydrothieno-1,4-dioxin)-poly (styrenesulfonate)), P3HT (poly(3-hexylthiophene-2,5-diyil)), MDMO-PPV (poly[2-methoxy-5-(3’,7’-dimethyloctyloxy)-1,4- phenylenevinylene]), PCBM ([6,6]-phenyl C61 butyric acid methyl ester), 1-2-4-trichlorobenzene, acetone, chlorobenzene, and isopropyl alcohol were purchased from Sigma-Aldrich and used without further purification.

### 2.3. Polymer Devices Fabrication Method

Ferrer et al. explained that organic photodetectors based on P3HT had a positive photocurrent response when the devices were illuminated by an intermittent light source [16]. This electrical characteristic makes them a good candidate for our research. Therefore, in our study, OPDs based on P3HT were used as receptors.

**Figure 4:**
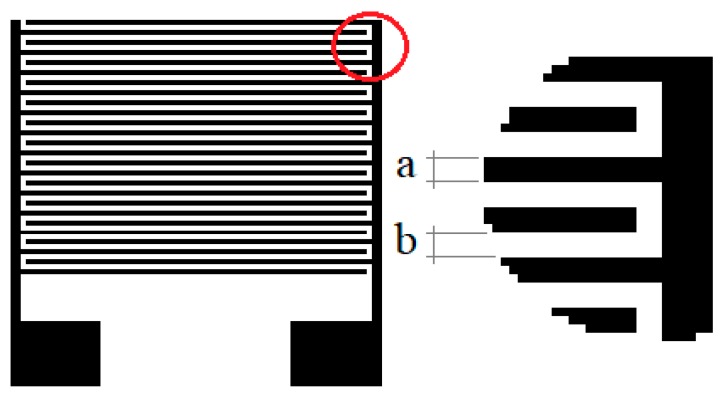
Schematics of the interdigital electrodes, where a is the finger width and b is the finger.

These devices are interdigitated electrodes. They are composed of a silicon substrate with a layer of silicon oxide with a 1.5-µm thickness. On the substrate, a 100 nm gold layer was deposited by evaporation, with a determined layout.In a single substrate, four different layouts were manufactured. Each layout depends on the thickness and separation of the gold fingers (Table 2). Due to this geometric variation, we could observe the influence of finger width on the electrical properties of the polymer layer, shown in Figure 4.

As Ferrer et al. indicated, in our study, the active layer was composed of P3HT. This layer was casted on the interdigitated electrodes using a spin coating technique. P3HT was dissolved in chlorobenzene with a 10 mg/mL of concentration. It was spin coated at 3000 rpm. Later, it was dried for 60 mins at 80 °C to evaporate the solvent residues.

### 2.4. Organic Photodetectors Devices Fabrication

As we have commented previously, organic photodetectors have been employed as receivers. The technique used the OPD manufacturing was the use of thin film deposition by spin-coating. The organic device structures were in the form of ITO/PEDOT:PSS/active layer/Al.

The substrate was commercial glass with a thin layer of indium tin oxide (ITO). They were cleaned by ultrasonic chemical baths. Sequentially, they were submerged in 1-2-4-trichlorobenzene, acetone, and isopropyl alcohol, for 20 mins in each, then dried with N_2_.

The PEDOT:PSS was spin-coated at 6000 rpm onto a glass-ITO substrate. In all organic receptors, PEDOT:PSS was employed as the hole transporting layer. Then, the PEDOT:PSS was dried at 120 °C for one hour. P3HT and a P3HT:PCBM blend (1:1 mass ratio) were used as the active layer. They were dissolved in chlorobenzene at 10 mg/mL. The active layers were spin-coated at 500 rpm and dried at 80 °C for one hour.

Finally, metallization was performed employing aluminium. It was evaporated in a high vacuum chamber until the layer reached 200 nm. After the metallization, an annealing ramp process was started. The devices were heated up to 120 °C. When the temperature was reached, it was saved for two mins. Subsequently, the devices were allowed to cool to room temperature.

### 2.5. Characterization

For organic photodetector device characterization, a Keithley 2400 Sourcemeter was employed. Besides, a xenon arc lamp and a AM1.5G filter were used to simulate the light conditions. The OPDs were electronically characterized both under 1 sun light conditions (100 mW/cm^2^, AM1.5G, 25 °C) and darkness.

**Figure 5:**
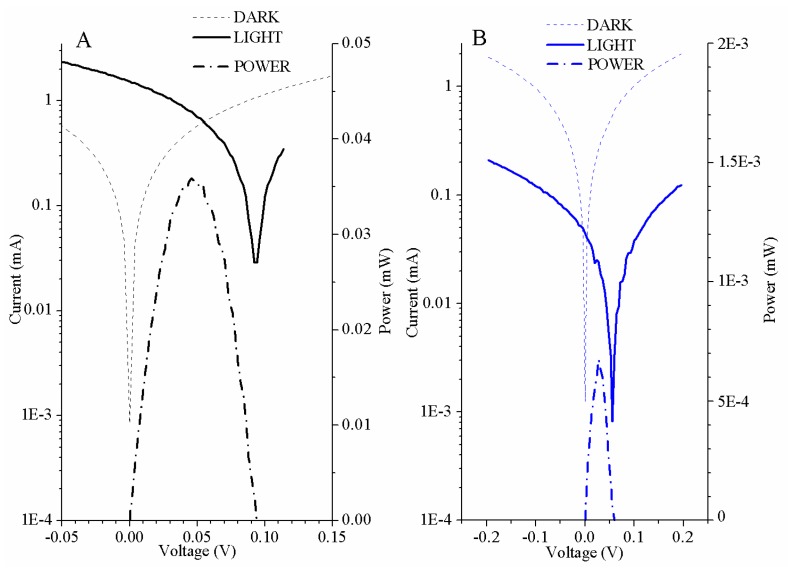
Electronic characterization of the organic photodetectors. Current versus voltage and power versus voltage curves in light conditions and in darkness for OPD based on poly(3-hexylthiophene):phenyl-C61-butyric acid methyl ester (P3HT:PCBM) (**a**) and for the OPD based on P3HT (**b**).

The characteristic parameters of solar cells are the short circuit current (*I_SC_*) and the open circuit voltage (*V_OC_*). In addition, the fill factor and the power conversion efficiency (from now on, efficiency) can be calculated, by the following expressions.

(1) $$FF=V_{MPP}I_{MPP}/V_{OC}I_{SC}$$

(2) $$\eta =P_{out}/P_{in}$$

where *V_MPP_* and *I_MPP_* are the voltage and current of the maximum power point. The efficiency is the relation between the maximum power generated by the solar cell and the incident power radiation. In our study, the power radiation was 0.4 W, due to the collection surface (4 cm^2^) and the light conditions. The data collection in the measurements (Figure 5) showed that the OPD based on the polymer blend present improvements in all electrical parameters. Although, the fill factor is slightly higher in the OPD based on pristine P3HT. In these kinds of devices, it is not common to use a P3HT polymer as an active layer, because the electrical characteristics have very low values, as Table 3 indicates.

Another crucial characteristic parameter of a photodetector is the external quantum efficiency (EQE), defined as the number of generated electrons per number of incident photons, without taking into account reflection losses. Sometimes, it could also be read as incident photons to current efficiency (IPCE). Under monochromatic light illumination, this is defined by Equation (3):

(3) EQE(λ)≡IPCE(λ)=Isc(λ)eP0(λ)hcλ=hceλIsc(λ)P0(λ)≈(1240·V·nmλ[nm])(Isc(λ)[mA]P0(λ)[mW])

where *I_sc_* is the short-circuit current, *P*_0_ the power of incident light, *h* is Planck’s constant, *c* the velocity of light, *λ* is the light wavelength, and *e* is the electron charge. The unit inside the brackets in the last term of the Equation (3) represents just a calculation suggestion, and the use of any other units is welcome, provided the overall equation is dimensionless.

A high value of EQE does not guarantee good photovoltaic energy conversion, however, it is essential. The quantum conversion efficiency of solar cells is usually much lower than 100%, due to losses associated with the reflection of incident photons, their imperfect absorption by the photoactive material, and the recombination of the charge carriers before they reach the electrodes. Furthermore, there are electrical resistance losses in the device and in the external circuit.

Concerning the EQE measurement of our device, it has been performed with the aid of a silicon calibrated photodiode, namely, model S2281 from Hamamatsu Photonics, for which its EQE is well known. In order to eliminate the influence of the incident light, both measures of the photocurrent of the Si-calibrated photodiode and our photodetector have been carried out for each wavelength and under the same light conditions. Dividing Equation (3), which was evaluated for our device by the same equation for the Si photodiode, allows the obtainment of the ratio of EQEs in terms of the ratio of photocurrents, as show in Equation (4):

(4) $$EQE_{CELL}\left(\lambda \right)=\frac{I_{sc}^{\left(CELL\right)}\left(\lambda \right)}{I_{sc}^{\left(Si\right)}\left(\lambda \right)}EQE_{Si}\left(\lambda \right)$$

where the subscript and superscript CELL and Si stand for our detector and the Si-calibrated photodiode, respectively.

**Figure 6:**
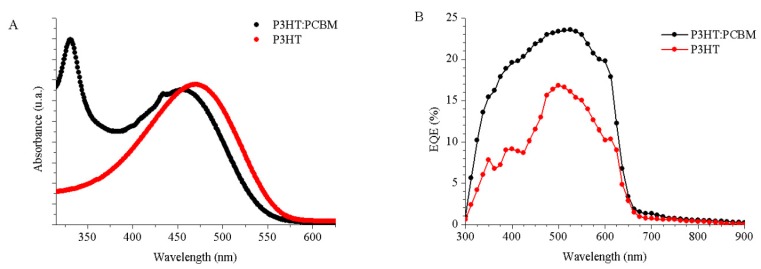
Optical absorption spectra (**a**) and external quantum efficiencies (**b**) for the OPD based on P3HT:PCBM (black circle) and for the OPD based on P3HT (red circle).

Figure 6 shows the optical absorption (Figure 6a) and the external quantum efficiency (Figure 6b) of the photodetectors used in our system. The optical absorption of the P3HT layer has a peak at 475 nm. This peak is characteristic of the P3HT polymer [17]. The absorption spectrum of P3HT:PCBM shows the absorption of the P3HT polymer but with a slight redshift at ~450 nm. Furthermore, the contribution of the PCBM polymer in the blend is observed in the spectrum at ~325 nm. Regarding the external quantum efficiency of the devices, the photodetector based on P3HT:PCBM is higher than the EQE of the P3HT OPD. Both EQE curves have a good agreement with the absorption spectra, respectively.

In the response speed of the overall transmitter-receiver system, each element of the system has its own time response. Therefore, the total response, *t_Total_*, of the communication system could be described in terms of the contribution of each part, as summarized in Equation (5). That is, the transmitter (LED) with a time constant *t_LED_* and the receiver, which has the contribution of the organic photodetector, *t_OPD_*, and the circuitry in charge of collecting the current, which, in our case, was carried out by means of a 1 kΩ resistor and an oscilloscope, *t_OSC_*.

(5) $$t_{Total}^{2}=t_{LED}^{2}+t_{OPD}^{2}+t_{OSC}^{2}$$

On the other hand, assuming that each element has a low-pass frequency response with a simple pole and by defining the rise time of a signal as the measure of the time response to a stepped or Heaviside function, in a such a way that the time for the output changes from 10% to 90% of the steady output level, a relationship between the rise time and the bandwidth of the signal can be easily derived, as shown in Equation (6):

(6) $$t_{rise}=\frac{0.35}{f_{3\mathrm{dB}}}\Leftrightarrow \mathrm{BW}=\frac{0.35}{t_{rise}}$$

Coming back to the whole-time response of the communication system, our bottleneck comes from our OPD, because the oscilloscope used to characterize the signal has a 100 MHz bandwidth, and also the commercial LEDs used are wide enough in comparison to the organic photodetector bandwidth.

At this point, a characterization of the OPD rise time of the electrical signal to a stepped optical pulsed has been performed for each LED, as this rise time depends on the incident light wavelength and load resistance. The data are summarized in Table 4. As a RGB LED sample, the green-LED was used.

The time response of the OPD, in turn, is determined by the following factors, namely, the terminal capacitance and load resistance (*t*_1_), the diffusion time of carriers generated outside the depletion layer (*t*_2_), and the carrier transit time in the depletion layer (*t*_3_).

So, the organic photodetector time *t_OPD_* could be described by Equation (7), which is totally analogous to Equation (5).

(7) $$t_{OPD}=\sqrt{t_{1}^{2}+t_{2}^{2}+t_{3}^{2}}$$

Finally, the photo sensitivity of the OPD has been evaluated from its external quantum efficiency. The calculation of this datum is straightforward, with no more than clearing than the last term on the second half of Equation (3), leading to Equation (8):

(8) $$S_{OPD}\equiv \frac{I_{SC}\left(\lambda \right)\left[A\right]}{P_{0}\left(\lambda \right)\left[W\right]}=\frac{\lambda \left[\mathrm{nm}\right]}{1240\cdot V\cdot \mathrm{nm}}\mathrm{EQE}\left(\lambda \right)$$

**Figure 7:**
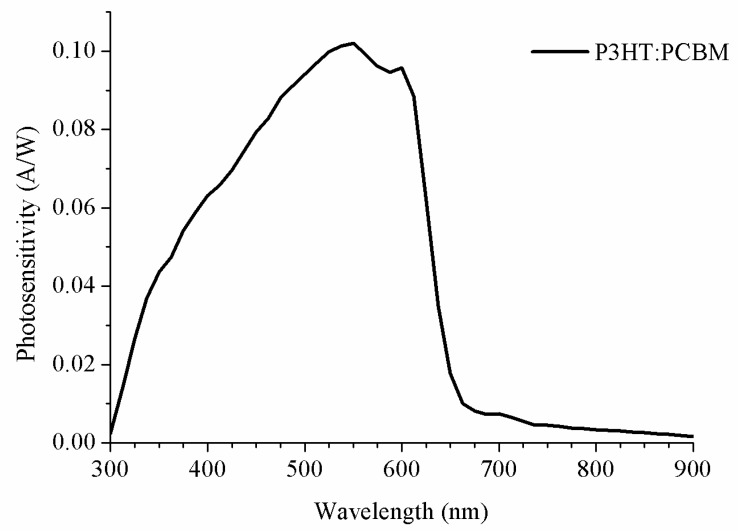
Results of photosensitivity for the OPD based on P3HT:PCBM.

With the OPD photosensitivity, Figure 7, we can calculate the current produced by each LED used in the photodetector. For a blue LED, this was 0.087 A/W, for a green LED, this was 0.095 A/W, for red LED, this was 0.023 A/W, and for a HL-White LED, this resulted in 0.084 A/W and 0.099 A/W for each emission peak, and for a LL-White LED, this resulted in 0.082 A/W and 0.100 A/W for each peak. 

The data collection of our system was carried out using an arbitrary waveform generator (AWG, Tektronix ^®^ AFG320) with a real-time oscilloscope (RTO, Tektronix ^®^ TDS3012).

## 3. Results

**Figure 8:**
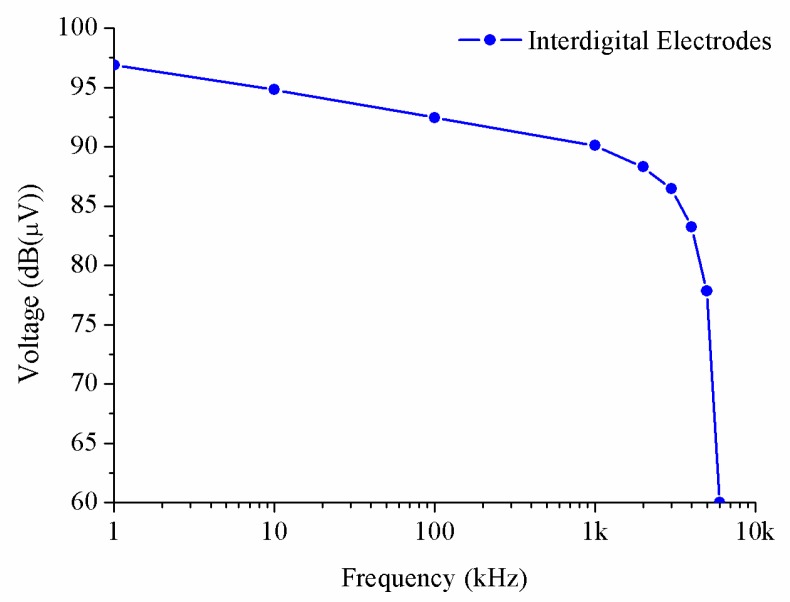
Results of maximum supported frequency of the system using interdigital electrodes.

In order to explore the bandwidth limitations of the system, we have used a periodic sinusoidal signal and we have increased the frequency and recording the variation of the amplitude until obtaining a fall of 3 dB with respect to the maximum. We have measured this curve with an input resistance of 1 MΩ. The obtained results in Figure 8 show the maximum supported frequency of the system using interdigital electrodes and they were better than we had expected. During the experiment, the single substrate was electromagnetically isolated, but the results are so disruptive that it is not possible to compare one with another. For this reason, the rest of the tests were done using different polymer layers. 

**Figure 9:**
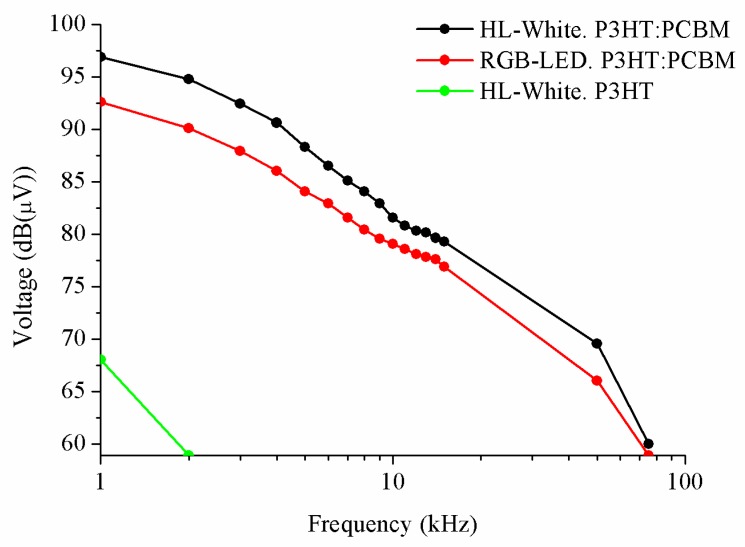
Results of maximum supported frequency of the system using P3HT:PCBM and P3HT.

The results of Figure 9 show the maximum supported frequency of the system using P3HT-PCBM and P3HT using different LEDs. In the case of red, green, and blue LEDs, we only show one curve in order to represent the results because the results of three ones are equal. The different bandwidths of the devices may be due to the polymer blend, which has a higher absorption and a higher EQE. In addition, the P3HT:PCBM blend has a greater charge mobility than the P3HT polymer, [18], improving its bandwidth [19].

The tests were done with the same block diagram and the results are shown in Figure 1, using the AWG with an input voltage of 4 V in transmission. In these cases, the minimum level detected in reception using the RTO was 1 mV.

**Figure 10:**
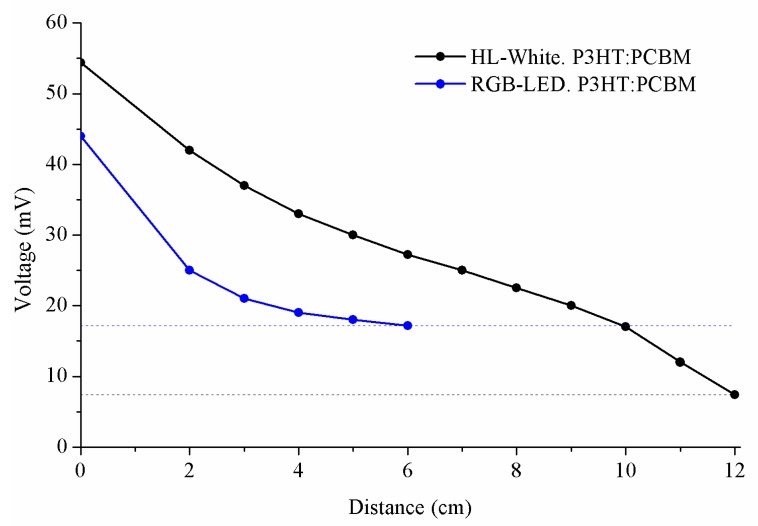
Results of maximum supported distance of the system using P3HT:PCBM and P3HT.

In these tests, the results were obtained with the same block diagram, shown in Figure 2, using an ATmega 328 microcontroller in order to transmit a frame based on Manchester coding with a frequency of 1 KHz. The reception of the tests was carried out using another ATmega 328 microcontroller and a computer to show the results. Note that in Figure 10, the minimum value of voltage to detect the text is different depending on the type of LED, where HL-White receives text correctly up until 7.4 mV, and in the case of LEDs with different colours (red, green, and blue) these receive text up until 17.2 mV, respectively. The information about the noise (shot noise or thermal noise) in the device could be obtained using the EQE graphs, electrical bandwidth, and photocurrent in the dark, and all of these properties have been shown in this text. Manchester coding type 1 was used without error detection code.

**Figure 11:**
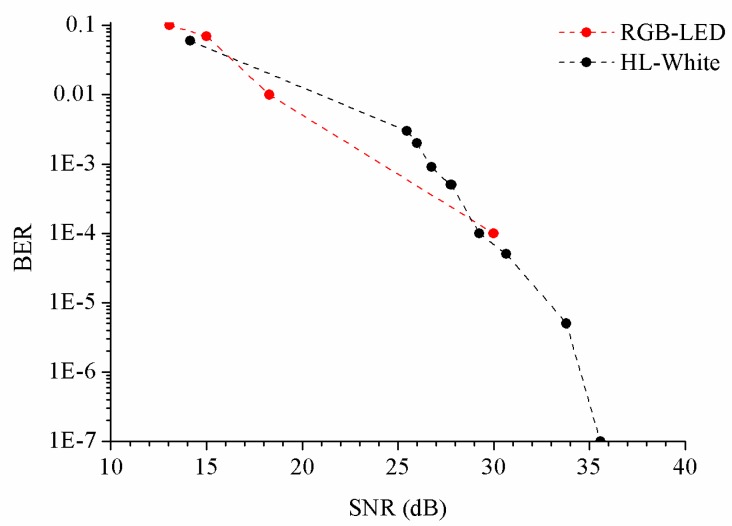
Results of BER versus signal-to-noise ratio (SNR) for the RGB-LED (red) and HL-White LED (black).

The Bit Error Rate (BER) was modified by the optical power. Therefore, the emitter-detector distance is a factor to consider, but it does not depend on the photodetector. However, BER versus distance measurements were performed for different LEDs, shown in Figure 11.

Our VLC system could be improved in different ways. On the one hand, regarding the photodetector, the efficiency of the devices increases under controlled ambient humidity conditions and when in an inert atmosphere [20]. Besides, improvements in the properties of the devices have been observed due to the encapsulation of the detectors [21]. To improve the bandwidth, nanoparticles could be included in the polymeric layers of photodetectors. Cadmium sulphide nanocrystals have been used to enhance the electron carrier mobility of devices [22].

On the other hand, regarding the communications system, one of the possibilities to improve the system is incorporating equalizers in reception in order to reduce the errors, as seen in [23].

We are studying possible improvements to our system, but the goal of our manuscript is to report the possibility of using organic photodetectors in VLC systems and take advantage of their ease of manufacturing and low cost.

## 4. Conclusions

In this paper, we have shown a low-cost system based on organic photodetectors using a ATmega 328 microcontroller and a frame based on Manchester coding type 1 to support an OWPAN. On the one hand, the system permits the transmission of text using an open-source electronic prototyping platform, commercial LED, and self-powered organic photodetectors, but has limitations concerning the frequency due to the use of these devices. On the other hand, we can increase the distance and frequency of transmission and reception by adding error detection code and using another electronic prototyping platform with a greater clock frequency, and the utilization of instructions of assembly code in order to optimize the source code, respectively. One of the improvements that we could implement would be to use an organic LED so that we obtain a fully organic system. An example of this can be found in [23]. In the case of organic photodetectors, a slow response time should be critical for this application, in which changes of light intensity are fast. However, in logistics, where storage management systems require the implementation of technological solutions that allow the control of merchandise in real time by means of LED signals that send information about the product, the data rate is not so important, and the use of these kinds of photodetectors could be an optimal solution. Additionally, properties of organic photodetectors can be improved by developing new materials with high charge mobility and through enhancements in manufacturing. Consequently, the response speed of organic devices may be increased.

## Figures and Tables

**Table 1:** Comparison between the different LEDs. Wavelength of the maximum emission peak and full width at half maximum (FWHM). LL: Low luminosity. HL: High luminosity.

LED	Blue	LL-White	HL-White	Green	Red
Emission Peak	472 nm	459 nm 559 nm	465 nm 562 nm	581 nm	646 nm
FWHM	28 nm	25 nm 116 nm	27 nm 120 nm	32 nm	43 nm
Irradiance with respect to BPW34	2.053 W/m^2^	2.053 W/m^2^	2.258 W/m^2^	2.053 W/m^2^	2.053 W/m^2^

**Table 2:** Fingers width (**a**) and separation (**b**) of the photodetectors.

Photodetectors	Fingers Width (a) µm	Fingers Separation (b) µm
50–50	50 µm	50 µm
5–20	5 µm	20 µm
10–40	10 µm	40 µm
25–25	25 µm	25 µm

**Table 3:** Summary of the electronic parameters of organic photodetectors.

	Short Circuit Current I_SC_ (mA)	Open Circuit Voltage V_OC_ (V)	Fill Factor FF (%)	Power Efficiency η (%)
P3HT	0.04 mA	0.06 V	26.62%	1.68·10^-4^%
P3HT:PCBM	1.52 mA	0.09 V	25.14%	0.01%

**Table 4:** Summarized OPD rise time and bandwidth for each LED.

LED	Rise Time (ms)	Bandwidth (kHz)
HL-White	0.080	4.375
Green	0.065	5.384
